# Trajectory of brain-derived amyloid beta in Alzheimer’s disease: where is it coming from and where is it going?

**DOI:** 10.1186/s40035-024-00434-9

**Published:** 2024-08-19

**Authors:** Ni Liu, Anaer Haziyihan, Wei Zhao, Yu Chen, Hongbo Chao

**Affiliations:** 1 Zhengzhou University, Zhengzhou, 450001 China; 2 Wuhan National Laboratory for Optoelectronics, Huazhong University of Science and Technology, Wuhan, 430074 China; 3 Huazhong University of Science and Technology, Wuhan, 430074 China

**Keywords:** Alzheimer’s disease, Amyloid-β, Periphery system, Immune clearance

## Abstract

Alzheimer’s disease (AD) is a progressive neurological disorder that primarily impacts cognitive function. Currently there are no disease-modifying treatments to stop or slow its progression. Recent studies have found that several peripheral and systemic abnormalities are associated with AD, and our understanding of how these alterations contribute to AD is becoming more apparent. In this review, we focuse on amyloid‑beta (Aβ), a major hallmark of AD, summarizing recent findings on the source of brain-derived Aβ and discussing where and how the brain-derived Aβ is cleared in vivo. Based on these findings, we propose future strategies for AD prevention and treatment, from a novel perspective on Aβ metabolism.

## Background

Alzheimer’s disease (AD) is an age-related neurodegenerative disease that can lead to brain atrophy and neuronal death in the brain [1]. AD is the most common type of dementia. In the 2019 Global Status Report on the Public Health Response to Dementia, the World Health Organization (WHO) highlighted that approximately 55 million patients worldwide are suffering from AD. Without significant medical breakthroughs in the prevention, slowing, and treatment of AD, the number would reach 150 million by 2050. However, after nearly a century of research, effective preventive strategies and treatments for AD are still lacking. A deeper understanding of the pathogenesis of AD may facilitate development of therapeutic strategies for AD.

AD pathogenesis is complex, involving extracellular amyloid-beta (Aβ) protein plaques, intracellular tau, neurofibrillary tangles, cholinergic insufficiency, oxidative stress, mitochondrial dysfunction, inflammation, and hormonal imbalances [2]. Brain Aβ plaques are the major pathological hallmark of AD [3]. Hardy et al. [4] found that amyloid precursor protein (APP) gene mutations can lead to massive Aβ deposition in the brain. They further proposed the Aβ cascade hypothesis, supported by the molecular genetic data of early-onset familial AD. The hypothesis states that when the amount of Aβ generated is greater than the amount that is degraded, accumulation occurs, leading to plaque formation, tau tangle formation, neuronal death, and a cascade of neuroinflammatory responses [5]. The anti-Aβ monoclonal antibodies Aducanumab and Lecanemab have been approved by the United States Food and Drug Administration (FDA) for brain Aβ clearance.

Both human and mouse studies have demonstrated that high levels of Aβ can flow from the brain to the periphery, and physiological catabolism of brain-derived Aβ can occur in the peripheral system [6]. This provides a novel perspective for understanding the pathogenesis of AD and developing therapeutics. In this review, we focus on the systemic role of Aβ in AD, discussing the major source of brain Aβ and associations of brain-derived Aβ aggregates with inflammation in the brain. We also discuss the communications between peripheral and central pools of Aβ, the peripheral pathways of brain-derived Aβ clearance, and how systemic diseases might interfere with brain-derived Aβ clearance. Finally, we summarize therapeutic strategies targeting Aβ.

## Where does brain-derived Aβ come from?

### The generation and spread of Aβ

**Fig. 1:**
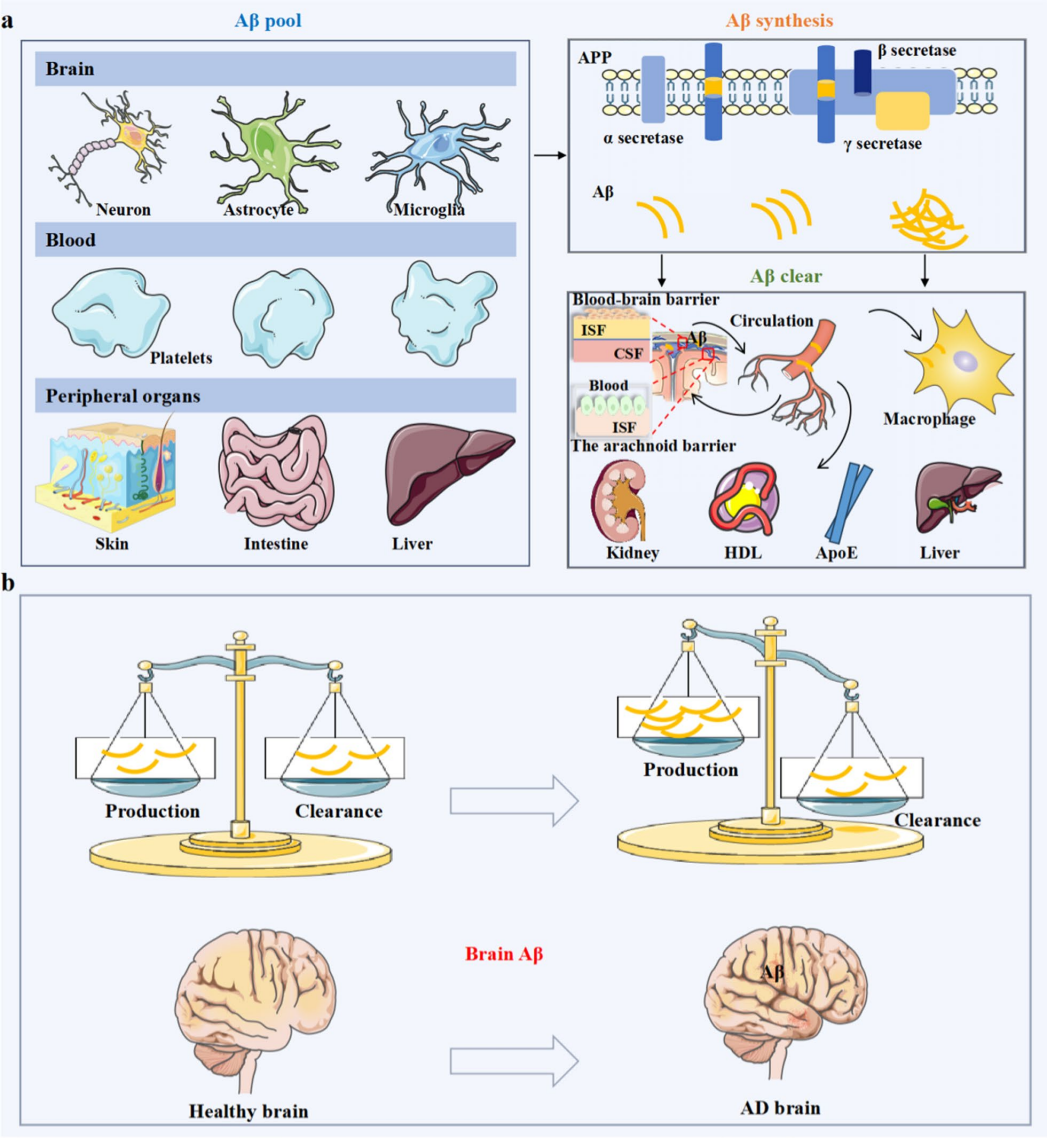
The generation and clearing path of Aβ. **a** The source, synthesis, and clearance of Aβ under normal physiological condition. Aβ is produced by neurons, microglia and astrocytes in the brain, platelets in the blood, and the skin, intestine, and liver in the periphery. Some Aβ is released from the brain to the blood via the blood–brain barrier (BBB), the interstitial fluid (ISF) bulk flow or cerebrospinal fluid (CSF) leakage. Aβ can also be transported to peripheral organs or tissues by carriers, where it is degraded by macrophages or hepatocytes or excreted via the liver or kidney. Under normal physiological conditions, high-density lipoprotein (HDL) prevents Aβ vascular accumulation independently of its primary receptor, scavenger receptor class B type 1 (SR-B1). ApoE promotes Aβ clearance by activating phagocytosis and increasing Aβ migration in microglia, where ApoE4 has a reduced capacity to induce these phenotypes than ApoE3. **b** Under normal physiological conditions, there is a balance between Aβ production and clearance (healthy brain without Aβ deposits). Under pathophysiological conditions, there is a loss of the balance between Aβ production and clearance, leading to Aβ aggregation in the brain (AD brain with Aβ deposition)

Aβ is a peptide of 38–43 amino acids generated from sequential cleavage of β-APP by β-secretase (BACE1) and γ-secretase [7]. BACE1 is an aspartyl protease that cleaves APP primarily at a single site, β-site1, whereas the γ-secretase complex cleaves the C-terminal fragment (CTF) at multiple sites, with preference for positions 40 and 42, forming the Aβ_40_ and Aβ_42_ peptides [8] (Fig. 1a). Previous studies have indicated that Aβ_38_ and Aβ_3–40_ are more amyloidogenic than other forms of Aβ, such as Aβ_4–40_ [8]. APP is a transmembrane protein widely expressed in brain neurons and various peripheral tissues, including blood (platelets) and peripheral organs (skin, intestines, and liver) [9, 10] (Fig. 1a). Alternative splicing of exons 7 and 8 of *APP* leads to generation of three major isoforms of APP, APP_695_, APP_751_, and APP_770_, comprising 695, 751 and 770 amino acids, respectively [11]. The APP_695_ isoform is predominantly expressed in neurons and lacks two exons. APP_751_ and APP_770_ possess a Kunitz-type serine protease inhibitor domain encoded by exon 7, while APP_770_ contains an additional immunoregulatory OX-2 antigen domain encoded by exon 8 in its extracellular region. Both APP_751_ and APP_770_ are expressed in both the brain and peripheral tissues [12, 13]. Therefore, AD is a disorder of both the central nervous system (CNS) and the peripheral system. Moreover, studies in animal models and cell cultures demonstrate that peripheral Aβ seeds accelerate plaque formation in a prion-like manner via templated seeding and intercellular propagation [14]. One study found that hepatogenic Aβ can enter the bloodstream through triglyceride-rich lipoproteins and further deposit in the brain to result in AD-like pathology, including neurodegeneration and brain atrophy [15]. Similarly, Aβ plaques have also been detected in the gastrointestinal tracts of AD patients and mice [16]. Additionally, when fluorescence-labeled Aβ is injected into the intestines of C57BL/6J mice, the fluorescence-labeled Aβ could spread to the hippocampus, resulting in brain disorder [17]. In another study, AD patient brain lysates were injected into the colons of wild-type (WT) mice, and induced AD-like pathology in the mice [18]. These studies suggest that Aβ in the gastrointestinal tract can also enter the brain parenchyma, resulting in Aβ deposition in the brain. However, it is still unclear how gastrointestinal tract Aβ is transmitted from the intestines to the brain and how Aβ deposition is induced.

Brain-derived Aβ can enter the peripheral blood through the blood–brain barrier (BBB) or cerebrospinal fluid (CSF) [9]. Given the bidirectional relationship between the brain and the periphery, the question arises concerning whether peripheral Aβ is the primary source of brain Aβ. Bu and colleagues used a parabiosis model between APPswe/PS1dE9 (APP/PS1) transgenic AD mice and their WT littermates, and found that the blood-derived Aβ can enter the brain and induce AD-like pathologies in WT mice, suggesting that the blood-derived Aβ may contribute to AD pathogenesis [19]. Lam and colleagues generated hepatocyte-specific human amyloid transgenic mice that produce pathogenic Aβ specifically in the liver. They found that the liver-derived Aβ travels through the circulatory system and crosses the BBB to enter the brain. The accumulation of Aβ in the brain drives the pathology associated with AD, including capillary dysfunction, inflammation and neurodegeneration [15]. Platelets are the primary source of Aβ in the peripheral circulation, and transplantation of bone marrow cells from APP/PS1 mice into WT mice leads to a continuous increase of human Aβ in the blood and Aβ deposition in the brain [20]. These findings suggest that AD is not simply a result of brain Aβ deposition.

### Aβ aggregation

Under normal physiological conditions, brain Aβ production and clearance are maintained at a dynamic balance and confer neuroprotection [9]. However, this balance can be disrupted by excessive production or decreased clearance of Aβ. Microglia dysfunction, low expression of low-density lipoprotein receptor-related protein-1 (LRP-1), apolipoprotein E (ApoE) overexpression, and ApoE binding to LPR-1 in competition with Aβ, can affect the clearance of Aβ [21, 22] (Fig. 1b). In addition, Aβ (especially Aβ_42_) is a hydrophobic peptide that is prone to aggregation. When Aβ_42_ exceeds a certain level, its monomers would aggregate into soluble oligomers, fibrils, and even insoluble plaques [23]. The brain Aβ_42_ patches will continuously recruit Aβ_42_ monomers and oligomers to form larger plaques [24]. Aβ oligomers are considered as the main neurotoxic components, and there is a negative correlation between the degree of oligomer aggregation and the level of toxicity [25]. The Osaka and Arctic mutations in *APP* can increase Aβ hydrophobicity, enabling aggregate formation [25, 26]. Studies have shown that the E693Delta *APP* mutation produces an Aβ variant more resistant to proteolytic degradation and showing enhanced oligomerization but no fibrillization, which is more likely to induce early symptoms of AD, indicating that the level of Aβ aggregates is closely related to the progression of AD [27].

## What harmful effects can brain-derived Aβ induce?

### Brain-derived Aβ aggregates induce polarization of microglia

**Fig. 2:**
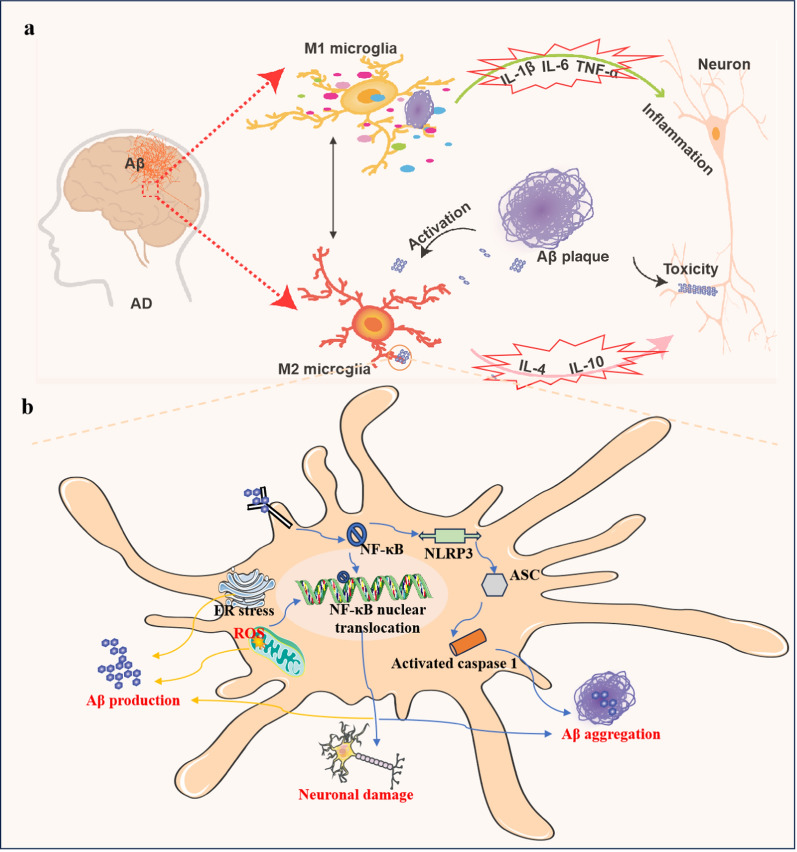
The hazards of brain Aβ aggregates. The diagram shows the dual functions of microglia. **a** Aβ aggregates promote polarization of microglia from M2 to M1 phenotype in the brain. The M1 microglia promote neuroinflammation through secretion of pro-inflammatory factors, leading to neuronal damage. **b** Aβ stimulates neuroinflammation, with involvement of nuclear factor-kappa B (NF-κB), endoplasmic reticulum (ER) stress, and the nucleotide-binding oligomerization domain-like receptor protein 3 (NLRP3) inflammasome pathway

Microglia are the primary immune cells in the brain and mainly include M1 and M2 microglia. The M1 type represents the pro-inflammatory phenotype, and M2 represents the anti-inflammatory phenotype [28]. M2 microglia can exhibit both alternative activation and acquired deactivation, which are induced by interleukin (IL)-4 and IL-10, respectively. In the early stage of AD, M2 microglia phagocytose misfolded Aβ and exert a neuroprotective effect [29] (Fig. 2a). However, when the Aβ plaque enrichment exceeds the capacity of microglial phagocytosis, the microglia would shift from the M2 to the M1 type, releasing a large amount of pro-inflammatory factors such as IL-1β and IL-6 [30, 31]. These pro-inflammatory factors can aggravate neuroinflammation, lead to neuronal damage, and further promote the production, aggregation, and deposition of Aβ, thus forming a vicious cycle [31–33] (Fig. 2a). The M1 microglia also highly express inducible nitric oxide synthase (iNOS), while reducing iNOS in APP/PS1 mice can reduce Aβ plaques in the brain [34]. In AD pathology, both disease-associated microglia and down-regulation of homeostasis-associated genes are related to the upregulation of AD-related genes, including *APOE*, triggering receptors expressed on myeloid cells-2 receptor2 (*TREM2*) and *TYROB* (tyrosine protein–protein binding protein) [3]. TREM2 is only expressed in microglia in the CNS and is a positive regulator of phagocytosis [35]. TREM2 is a receptor for lipoproteins, and mediates clearance of lipoprotein-Aβ complexes by microglia [36]. TREM2 has a dual influence on AD pathogenesis. It can form a neuroprotective barrier around Aβ aggregates and prevent their spreading outward, resulting in dense Aβ plaques in the early stage of AD. On the other hand, TREM2 also promotes inflammation in the middle and the late stages of AD [31, 37, 38].

In addition, the nuclear factor κB (NF-κB)/C3/C3aR signaling can regulate Aβ aggregation through astrocytes and microglia [35]. Brain Aβ aggregates activate the NF-κB signaling in astrocytes, which release the complement protein C3a [35]. The interaction of C3a with C3a receptors on microglia and neurons promotes Aβ aggregation, and blocking the interaction between C3a and microglia can reduce microglial activation and the Aβ load in the brain [39].

### Brain-derived Aβ aggregates stimulate neuroinflammation

Neuroinflammation is mediated mainly by microglia and is one of the main causes of neuronal damage and necrosis [35]. Astrocytes also play an auxiliary role in neuroinflammation. Early misfolding and aggregation of Aβ in the brain can be cleared by binding to receptors on the surface of microglia, such as receptors for advanced glycation end product, Toll-like receptor (TLR), nucleotide-binding oligomerization domain-like receptor protein 3 (NLRP3), and formyl peptide receptor (Fig. 2b) [40]. Loss of TLR4 and NLRP3 inflammasome in mice can reduce Aβ accumulation and production of pro-inflammatory cytokines IL-1β and IL-18 [41]. Additionally, when NLRP3 is activated in response to damage-associated molecular patterns (DAMPs) or pathogen-associated molecular patterns (PAMPs), it recruits ASC (apoptosis-associated speck-like protein containing a caspase recruitment domain [CARD]) through PYD-PYD (Pyrin domain) interactions, leading to the formation of active caspase-1 through CARD-CARD interactions. NLRP3 activation exacerbates the accumulation of Aβ (Fig. 2b) [42], and Aβ aggregates would, in turn, promote the generation of IL, mitogen-activated protein kinase, NF-κB and reactive oxygen species (ROS), thus aggravating neuroinflammation [34, 43, 44]. ROS production has also been identified as a mediator of Aβ-induced endoplasmic reticulum (ER) stress and cytotoxicity [45]. Moreover, ER stress contributes to the progression of AD pathology [46] (Fig. 2b).

Neuroinflammation can also result in the engulfment of misfolded proteins such as Aβ by microglia and astrocytes [47]. Although controversy exists regarding the order of occurrence of Aβ aggregation and neuroinflammation, it is relatively clear that Aβ aggregation accelerates the development of neuroinflammation, which in turn promotes the production and aggregation of Aβ [48]. Neuroinflammation is a chronic response of the innate immune system, resulting in the failure of Aβ clearance.

## How can brain-derived Aβ be cleared?

### Aβ clearance strategies from the brain to the periphery

Lack of Aβ clearance is considered the main cause of Aβ deposition in patients with sporadic AD [49]. Therefore, accelerating the clearance of Aβ is a promising strategy for treating AD. Studies using the deep cervical lymph node ligation method in AD mice have revealed that brain lymphatic clearance is a physiological mechanism for Aβ transport from the brain to the periphery [50].

Brain-derived Aβ has been detected in the periphery of AD patients and AD mice. Aβ flows from the brain to the periphery mainly through the BBB pathway and the brain lymphatic pathway (Fig. 1a). In the BBB pathway, the capillary lengths in mouse and human brains are approximately 0.6 km and 650 km, respectively, which account for more than 85% of the total length of cerebral blood vessels, providing a large surface area for substance exchange between the blood and the brain [24, 51]. Brain-derived Aβ efflux is usually mediated by brain endothelial cell surface proteins, mainly LRP-1 [52]. Previous studies have suggested that approximately 40%–60% of brain-derived Aβ is cleared in the periphery. Aβ clearance via the BBB is reduced by approximately 30% in AD patients [53, 54]. The brain lymphatic pathways include the perivascular pathway, glymphatic system, meningeal lymphatic vessels, and olfactory/cervical lymphatic drainage, which drains Aβ in the CSF to the deep cervical lymph nodes, allowing Aβ and other macromolecules to drain from the brain to the periphery [27]. The exact contribution of each mechanism to the overall Aβ clearance remains unclear. Nevertheless, these pathways work together to remove pathological proteins from the brain to the periphery. These findings suggest that peripheral tissues and organs are physiologically involved in the clearance of brain-derived Aβ, including the blood, liver, kidney, spleen, and gut (Table 1).

**Table 1:** Strategies for Aβ clearance via the peripheral system

Peripheral system	Mechanisms	Strategy	Research findings	References
Blood	Enzymatic degradation	Insulin-degrading enzyme (IDE)	Astrocyte-secreted IDE regulates Aβ levels; a catalytically inactive IDE aids in Aβ degradation	[129, 142, 143]
		Neprilysin	Reduced neprilysin activity elevates Aβ oligomers, impacting cognitive function; neprilysin administration clears plasma Aβ	[57, 144, 145]
		Angiotensin-converting enzyme (ACE)	ACE inhibitor ramipril boosted Aβ in AD⁺ACE mice; intranasal ACE inhibitors mitigated inflammation in 5×FAD mice	[146, 147]
	Cell transplantation	Bone marrow	Bone marrow exosomes benefit AD behavior; young marrow preserves memory in mice	[55, 148]
		Bone marrow-derived cells	Bone marrow-derived cells recruit microglia in response to Aβ, contributing to cognitive improvement in AD	[44, 149, 150]
	Immune clearance	Monocytes	Aβ uptake by blood monocytes is reduced with aging in AD patients and mice	[67, 151]
		Macrophages	SHARPIN (Shank-associated RH domain-interacting protein) regulates Aβ phagocytosis and inflammation in macrophages; anti-oxidants could enhance the Aβ phagocytic efficacy of macrophages	[152, 153]
	Blood exchange	Hemodialysis	Dialysis effectively reduces Aβ_42_ and Aβ_40_ plasma levels in cognitively impaired patients	[154–156]
		Peritoneal dialysis	Peritoneal dialysis reduces Aβ plasma levels in humans and attenuates AD-associated phenotypes in APP/PS1 mice	[157]
		Whole blood therapy	Plasma exchange reduced Aβ in the plasma and decreased Aβ deposition in the cortex and hippocampus in APP/PS1 mice	[63, 158, 159]
Liver	Receptor regulation	Gene regulation	Enhancing hepatic Aβ clearance via LRP-1 overexpression attenuated cerebral Aβ deposition and cognitive impairments in APP/PS1 mice	[63]
		Enzyme activity	Liver X receptor agonist treatment prevented memory deterioration and significantly reduced hippocampal Aβ. Hepatic soluble epoxide hydrolase regulates cerebral Aβ metabolism	[160, 161]
	Peripheral sink	Liver enrichment	CuxO@EM-K reduces Aβ burden in the blood and brain	[64]
Kidney	Increase urination volume	Diuretic	Chronic furosemide treatment reduced blood and brain Aβ levels and attenuated AD pathologies and cognitive deficits in APP/PS1 mice	[66]
Spleen	Peripheral sink	Monocytes/macrophages	Spleen clears Aβ physiologically; splenectomy worsens AD-type pathogenesis	[68]
Gut	Inflammation regulation	Microbial diversity	Treatment with *Bifidobacteria* or GV-971 can suppress Aβ accumulation and neuroinflammation	[73, 74, 162]

#### Blood-mediated Aβ clearance

The peripheral sink hypothesis suggests that by increasing Aβ clearance from the blood, a concentration gradient is created, which favors the efflux of Aβ from the brain to the bloodstream, thus facilitating Aβ clearance [27, 55]. As blood components, secreted enzymes are critical for the catabolism of peripheral Aβ. These enzymes include insulin-degrading enzyme [56], neprilysin [57] and angiotensin-converting enzyme [58]. They have an affinity for specific domains of Aβ proteins and can cleave these peptides into shorter, more benign forms. Immune cells in the blood, such as monocytes and macrophages, can recognize Aβ and clear Aβ peptides from circulation. Supplementing blood components by means of bone marrow transplantation [55] or by administering bone marrow-derived cells [44] or albumin [59] can enhance the clearance of peripheral Aβ. In addition, peripheral Aβ can be cleared by blood exchange, including hemodialysis, peritoneal dialysis, and replacement of fresh blood (whole blood therapy) [60].

#### Liver-mediated Aβ clearance

The liver plays a crucial role in systemic metabolism and detoxification, including the clearance of various substances from the blood. A study found that approximately 13.9% of Aβ_42_ and 8.9% of Aβ_40_ are removed by the liver and that LRP-1 mediates the peripheral clearance of Aβ in the liver. LRP-1 overexpression in hepatocytes enhances hepatic Aβ clearance, decreases cerebral Aβ deposition and attenuates cognitive impairment in AD mice [61–63]. Moreover, self-protecting biomimetic nanozymes, such as CuxO@EM-K, have been designed to test the efficacy of therapies that can enhance the peripheral sink effect, which could potentially redirect Aβ from the brain to the liver for clearance [64].

#### Kidney-mediated Aβ clearance

The kidney is thought to be responsible for Aβ clearance by filtering Aβ from the blood to the urine. There is a positive association between chronic kidney disease and a risk of cognitive impairment [6, 65]. Both acute kidney ligation and unilateral nephrectomy aggravate Aβ burdening in the blood and brain, neuroinflammation, and neurodegeneration in APP/PS1 mice. Moreover, chronic furosemide treatment can decrease brain Aβ deposition and ameliorate cognitive deficits in AD mice [66].

#### Spleen-mediated Aβ clearance

The spleen acts as a blood filter and an immune organ. The mononuclear phagocyte system found primarily in the spleen, has been thought to contribute to the clearance (engulfing and digesting) of Aβ. Splenectomy reduces the monocyte-derived periplaque macrophages and the circulating myeloid cells, and worsens amyloid pathology in APP/PS1 mice [67]. In addition, Aβ levels in the splenic artery are higher than those in the splenic vein, suggesting Aβ clearance when blood flows through the spleen. Splenectomy aggravates AD-related behaviour deficits and pathology in AD mice [68]. Therapies aimed at enhancing Aβ clearance through the spleen and other peripheral organs are a topic of interest. However, further studies are required to comprehensively understand the role of the spleen in Aβ clearance, especially the precise interplay between the peripheral immune system and brain pathology.

#### Gut-mediated Aβ clearance

**Fig. 3:**
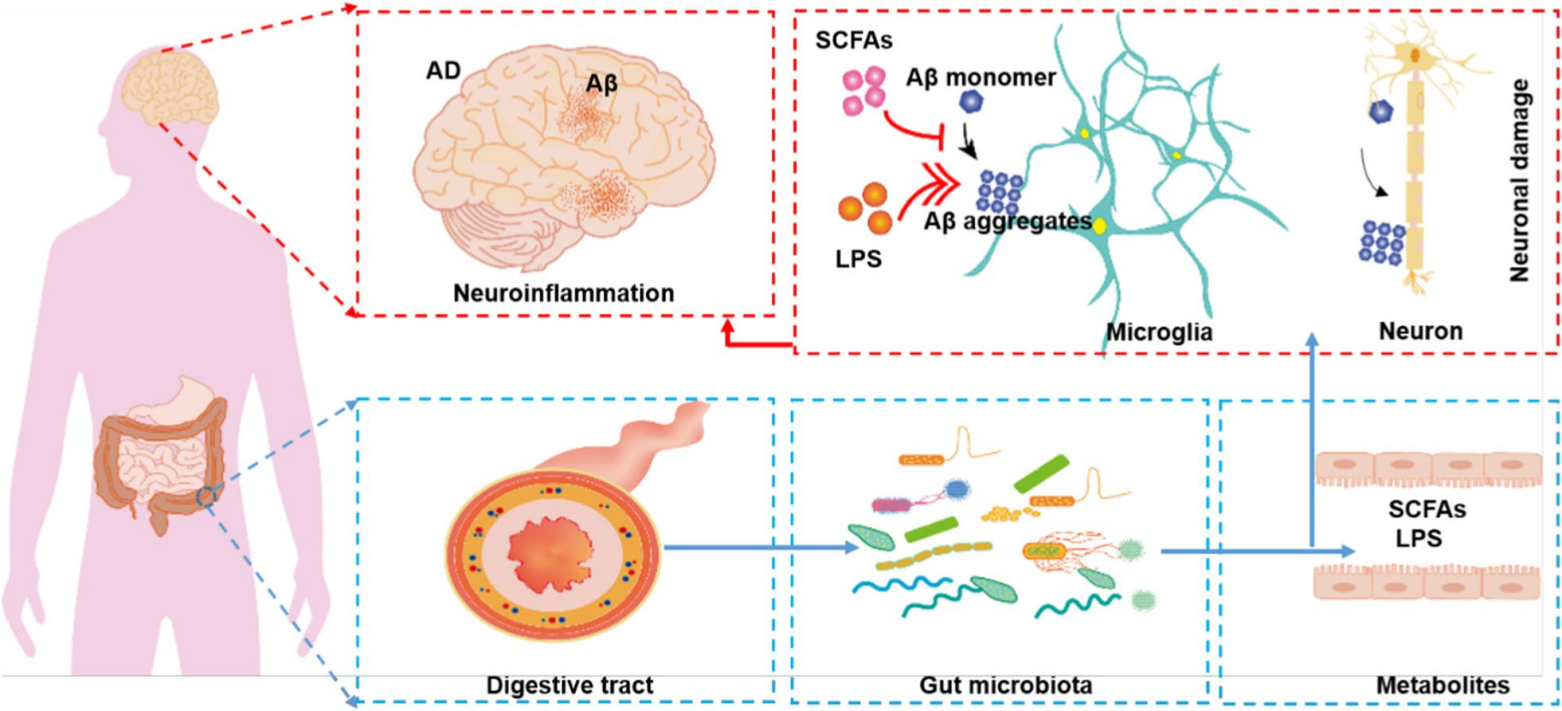
The secondary metabolite lipopolysaccharides (LPS) from the gut microbiota, can affect brain Aβ via the gut-brain axis, causing neuronal damage and local inflammation, and promoting local and systemic Aβ deposition. Short-chain fatty acids (SCFAs) from the gut microbiota can enter the bloodstream and maintain BBB homeostasis, inhibiting Aβ deposition.

Patients with AD often experience an imbalance in gut microbiota, such as an increase in pro-inflammatory gut microbes (e.g., *Shigella*) and a decrease in anti-inflammatory gut microbes (e.g., *Bifidobacterium*) [69]. This imbalance leads to abnormal secretion of secondary metabolites of gut microbes, such as lipopolysaccharides (LPS) and short-chain fatty acids (SCFAs). LPS acts on the TLR4-CD14/TLR2 receptors on leukocytes and microglia, leading to NF-κB-mediated increases of cytokines, resulting in elevated Aβ levels, damage to oligodendrocytes, and finally neuronal damage in AD brains [70]. In contrast, SCFAs, which are produced mainly by *Clostridium*, *Lactobacillus*, and *Bifidobacterium*, can cross the BBB via monocarboxylate transporters [71]. They can accelerate the clearance of brain Aβ by regulating the proliferation and differentiation of microglia. They can also directly bind to Aβ to inhibit the formation of Aβ plaques [72, 73]. Therefore, supplementation with probiotics can decrease the brain Aβ burden and ameliorate cognitive impairment in AD mice [66] (Fig. 3). In addition, the gut microflora in 5 × FAD and APP/PS1 mice facilitate entry of peripheral immune cells into the brain, leading to microglial activation and disease progression. Treatment with GV-971 in these mice can restore the gut microbial profile, ameliorate brain immune cell infiltration and inflammation [74], reduce brain Aβ burden and tau hyperphosphorylation, and improve cognitive function [74]. These studies demonstrated that the brain–microbiota axis is involved in the pathogenesis of AD, and it is speculated that the gut has the potential to regulate peripheral Aβ clearance [75, 76].

### Representative strategies for targeting Aβ clearance

**Fig. 4:**
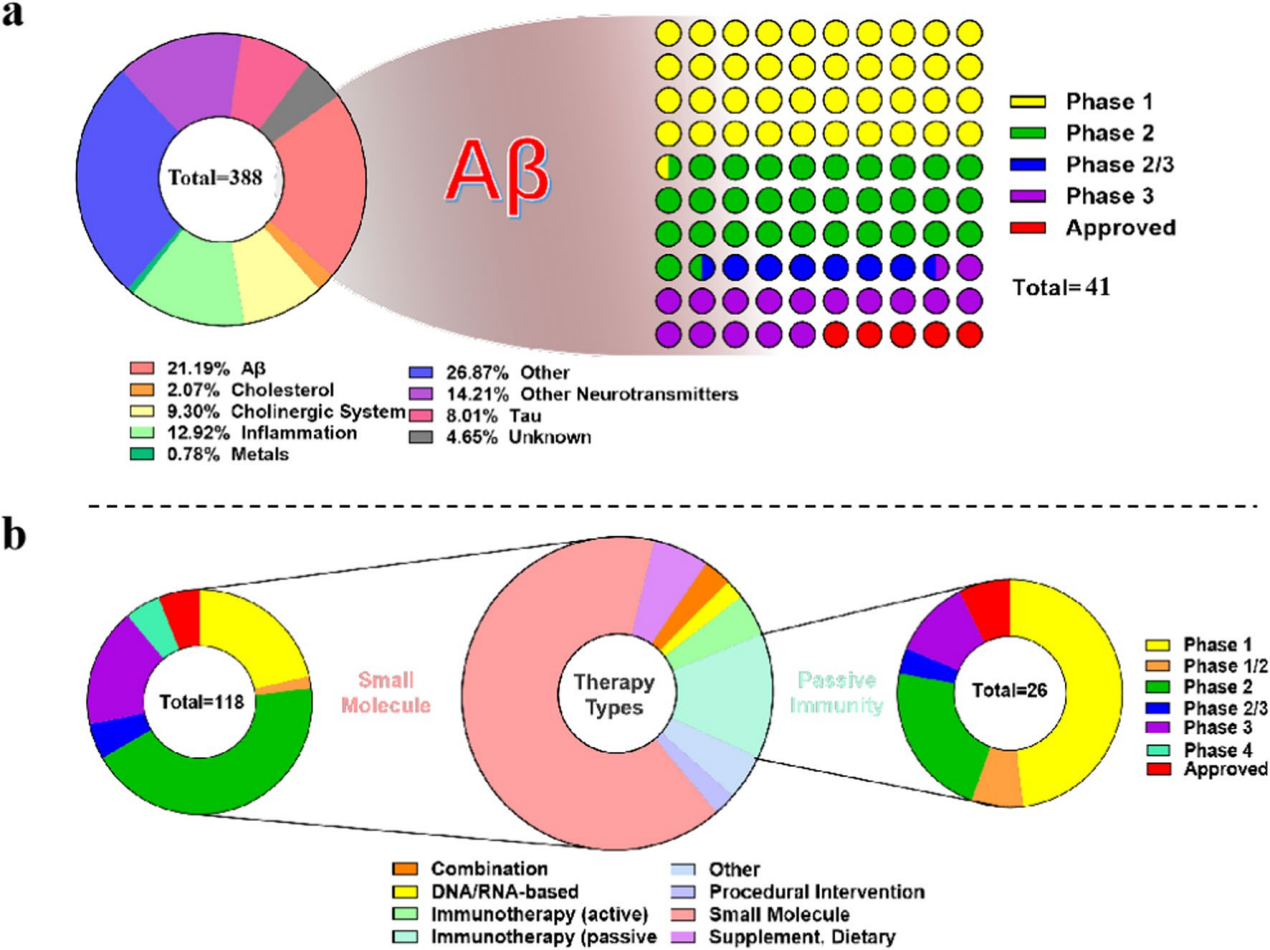
Therapeutic targets and therapy types for AD (https://www.alzforum.org/therapeutics. Accessed March 28, 2024). **a** There are 388 drugs in development for AD. They are categorized into nine target classes. Of the 82 drugs targeting Aβ, only 39 are under clinical validation and 2 have obtained FDA approval. **b** The 388 drugs are categorized into eight therapy types. Among them, 118 small-molecule drugs and 27 passive immunotherapy drugs are in clinical validation or have obtained FDA approval

In the past two decades, the development of AD drugs was particularly challenging. Most AD trials failed to demonstrate efficacy. However, confidence in the potential of AD drug development has not diminished [2]. In the Alzforum database, a total of 388 drugs in development for AD treatment are selected as eligible for scoring. These 388 drugs are categorized into eight therapy types and nine target classes. The largest number of drugs target Aβ (82, 21.19%), followed by those targeting inflammation (50, 12.92%), neurotransmitters and receptors (55, 14.21%), and tau (31, 8.01%) (Fig. 4a). At present, only 2 of the 82 Aβ-targeting drugs have been approved by FDA, with other 39 drugs undergoing clinical trials, 34 drugs having been discontinued, and 7 being inactive (Fig. 4a). Regarding the AD therapy type, most are classified as small-molecule drugs (222, 64.81%) or passive immunotherapy (44, 12.90%) (Fig. 4b). Among the 222 small-molecule drugs, seven have been approved by the FDA: galantamine, memantine, rivastigmine, suvorexant, brexpiprazole, donepezil, and tacrine. A further 110 drugs are undergoing clinical trials, 80 drugs have been discontinued or rejected, and only 24 are inactive. Moreover, among the 44 passive immunotherapy drugs, two have been approved by the FDA, i.e., Aducanumab and Lecanemab, for use as Aβ-clearing drugs, and 24 drugs are undergoing clinical trials (Fig. 4b). Therefore, Aβ-targeting drugs are an important focus of research attention. In the following, we will elaborate on small-molecule drugs and passive immunotherapy.

#### Small molecules for Aβ clearance

When considering small molecules as an Aβ clearance strategy in AD, the goal is to reduce Aβ peptide accumulation in the brain (Table 2).

**Table 2:** Representative small-molecule drugs for Aβ clearance (https://clinicaltrials.gov/; https://www.alzforum.org/therapeutics. Accessed January 28, 2024)

Agent	Clinical trial phase	Study population	Parameter	Adverse event	NCT identifier
Acitretin	II	Mild to moderate AD	MMSE	Gastrointestinal disorders	NCT01078168
ALZ-801	III	Early to mild AD	CDR-SB, MMSE	Mild nausea and vomiting	NCT04693520
ALZT-OP1	III	Early AD	CDR-SOB	Not specified	NCT02547818
Buntanetap	II	Mild AD	ADAS-Cog11, ADSC-CGIC	Dizziness, nausea, and vomiting	NCT02925650
CT1812	II	Mild to moderate AD	ADAS-Cog13, CDR-SOB	Encephalitis	NCT03493282
Lenalidomide	II	Mild cognitive impairment	ADAS-Cog, ADCS-ADL, CDR-SOB, MMSE	Not specified	NCT06177028
Levetiracetam	II	Patients with AD	ADCS-ADL, CDR	Somnolence	NCT01044758
Nasal Insulin	III	Mild AD	ADAS-Cog	Not specified	NCT02462161
NIC5-15	II	Mild to moderate AD	ADAS-Cog	Dizziness	NCT00470418
Varoglutamstat	II	Mild to moderate AD	ADNI	Not specified	NCT04498650

ADAS-cog, Alzheimer’s Disease Assessment Scale for cognition; ADCS-ADL, Alzheimer’s Disease Cooperative Study-Activities of Daily Living scale; CDR-SOB, Clinical Dementia Rating Sum of Boxes; ADAS-Cog 11, Alzheimer's Disease Assessment Scale-cognitive subscale 11; ADAS-Cog 13, Alzheimer’s Disease Assessment Scale-cognitive subscale 13; MMSE, Mini-Mental State Examination

##### Secretory enzyme regulators

The regulatory control of enzymes involved in Aβ secretion, such as BACE and γ-secretase, is an essential area for AD drug development. Drugs that regulate Aβ secretion include the β-secretase inhibitor Lanabecestat [77, 78] and the γ-secretase inhibitor Semagacestat [62], which can inhibit the BACE1 enzyme and the γ-secretase enzyme complex, respectively, further reducing Aβ production [62]. However, these drugs were discontinued in clinical trials because of ineffectiveness or liver toxicity. Several experimental small-molecule inhibitors and modulators targeting secretase enzymes and Aβ aggregation are under clinical trials or preclinical studies (Table 2). While these therapies offer promise, their development is complex, and identifying compounds that can effectively and safely regulate Aβ secretion remains a significant challenge.

##### Aβ aggregation inhibitors

Aβ aggregation inhibitors encompass many chemical structures, including small molecules and peptides that can bind to Aβ and interfere with its aggregation process. These compounds are designed to target specific regions of Aβ, such as the hydrophobic core involved in aggregation, to inhibit the formation of toxic Aβ oligomers and fibrils. The main Aβ small-molecule inhibitors that have entered clinical trials are 3-APS (III) [79] and 8-HQ (II/III) [80], both of which were discontinued because they did not consistently inhibit Aβ aggregation in the brain to improve cognitive performance in AD patients [81]. In contrast, ALZ-801 has shown good gastrointestinal absorption and stable plasma concentrations, with significant clinical effects in the *APOE4/4* high-risk population and dose-dependent preservation of the hippocampal volume [82]. Developing effective Aβ aggregation inhibitors is facing substantial challenges, including BBB penetration, target selectivity, and metabolic stability. Moreover, the potential off-target effects and the safety are crucial issues to be addressed.

##### Intestinal microbiota regulators

In the process of AD, the imbalance of gut microbiota leads to an abnormal increase in phenylalanine and isoleucine in peripheral blood, which in turn induces the differentiation and proliferation of peripheral pro-inflammatory Th1 cells and promotes their brain invasion [74]. Th1 cells infiltrating the brain and the intrinsic M1 microglia in the brain are activated together, leading to AD-related neuroinflammation [74]. Probiotics, prebiotics, synbiotics, and dietary interventions represent potential regulators of the intestinal microbiota, and their effects on Aβ metabolism and clearance have been investigated [83]. These interventions aim to modulate the composition and activity of the gut microbiota to promote a healthy microbial community and enhance Aβ clearance. However, perhaps because of the widespread use of oral probiotics as supplements, no probiotics are currently in clinical trials. Moreover, the strong acids and bile salts in the gastrointestinal tract may induce leakage of ions and other cellular components that may kill probiotics, resulting in their low efficacy or even ineffectiveness [73]. This poses a challenge to developing methods to effectively transport probiotics around the gastrointestinal barrier to the intestine so that they can exert their biological functions.

#### Passive immunotherapy for Aβ clearance

Anti-Aβ immunity includes active immunity and passive immunity. Active immunity strategies involve stimulating the immune system to produce anti-Aβ antibodies by administering Aβ or its fragments. This approach has the advantages of long duration and low cost, but adverse immune reactions are difficult to predict [84]. For example, the Aβ vaccines UB-311 and ACC-001 have been discontinued because they led to meningoencephalitis in AD patients [85, 86].

Passive immunotherapy accelerates Aβ clearance by peripheral injection of humanized anti-Aβ monoclonal immunoglobulins. Aβ passive immunotherapy mainly promotes Aβ clearance in the following three ways [87]. (1) The antibody directly binds to Aβ in peripheral blood to reduce the Aβ content in the blood. This leads to an imbalance of Aβ in the periphery and the brain, promotes the outflow of soluble Aβ from the CNS via LRP-1 expressed on the BBB, and indirectly reduces the Aβ load in the brain [88, 89]. (2) The antibody Fc segment binds to the Fc receptor on microglia, which stimulates microglial activation, promoting Aβ clearance [90, 91]. (3) The antibody acts directly on Aβ plaques, fibers, or oligomers in the brain after crossing the BBB, and then degrades and clears Aβ [92].

##### Targeting soluble Aβ

The aim of targeting soluble Aβ with antibodies is to prevent the aggregation of Aβ peptides into larger, more toxic oligomeric and fibrillar forms. By retaining Aβ in a soluble state, these antibodies may mitigate Aβ-induced neurotoxicity and reduce the formation of Aβ plaques. Several monoclonal antibodies targeting soluble Aβ have been developed and investigated in clinical trials. These antibodies are engineered to specifically recognize and bind to soluble Aβ peptides while avoiding binding to insoluble aggregates. The potential of these antibodies to slow the progression of AD by promoting Aβ clearance has been evaluated in clinical trials (Table 3). Solanezumab is a humanized monoclonal immunoglobulin G (IgG) antibody that recognizes the intermediate domain of Aβ peptides and can specifically recognize soluble Aβ monomers and prevent the formation of Aβ aggregates [93]. A clinical phase III study found that Solanezumab did not improve the cognition of AD patients using the Alzheimer's Disease Assessment Scale (ADAS), which can be used to evaluate the severity of dementia [94]. Lecanemab is a humanized IgG1 form of a mouse monoclonal antibody (mAb), mAb158, that can selectively bind large, soluble Aβ protofibrils and promote their clearance [92]. The results of the latest phase III clinical trial (1795 patients) also showed that compared with the placebo-treated group, the Clinical Dementia Rating Scale (CDR) sum of boxes scores of AD patients treated with Lecanemab for 18 months decreased by 27%, the decline in cognitive level decreased by 26%, and the decline in daily activity function decreased by 36% [95, 96]. Moreover, a significant decrease in Aβ deposition was observed in the brains of AD patients treated with Lecanemab.

**Table 3:** Representative Aβ antibodies for Aβ clearance (https://clinicaltrials.gov/; https://www.alzforum.org/therapeutics. Accessed January 28, 2024)

Agents	Clinical trial phase	Study population	Conformations recognized by antibody	Parameter	NCT identifier
ABBV-916	II	Early AD	N3pG-Aβ	PET, MRI	NCT05291234
ACU193	I	Mild AD	Soluble Aβ oligomers	MRI	NCT04931459
MEDI1814	I	Mild to moderate AD	Monomeric Aβ_42_	MRI, CSF total Aβ_42_	NCT02036645
Remternetug	III	Patients with AD	N3pG-Aβ	PET	NCT04451408
Solanezumab	III	Mild AD	Soluble Aβ monomeric	CDR-SOB, MMSE	NCT02760602
Trontinemab	I/II	Mild to moderate AD	Aβ fibrils	PET	NCT04639050
Leqembi	Approved	Early AD	Large, soluble Aβ protofibrils	ADAS-cog14	NCT03887455
Aducanumab	Approved	Mild AD	Aβ aggregates	CDR-SOB, MMSE, ADAS-Cog13, ADCS-ADL	NCT02484547

PET, positron emission tomography; MRI, magnetic resonance imaging; ADCS-ADL, Alzheimer’s Disease Cooperative Study-Activities of Daily Living scale; CDR-SOB, Clinical Dementia Rating Sum of Boxes; ADAS-Cog 14, Alzheimer's Disease Assessment Scale-cognitive subscale 14; ADAS-Cog 13, Alzheimer's Disease Assessment Scale-cognitive subscale 13; MMSE, Mini-Mental State Examination

##### Targeting insoluble Aβ

Monoclonal antibodies that target insoluble Aβ have been developed to specifically recognize and bind to insoluble Aβ aggregates. These antibodies are designed to facilitate the clearance of existing plaques through mechanisms such as opsonization and activation of microglia-mediated phagocytosis. These approaches seek to reduce the neurotoxic effects associated with Aβ aggregation and improve cognitive function in individuals with AD. Several monoclonal antibodies targeting insoluble Aβ have been investigated in clinical trials (Table 3). These trials have assessed the safety and efficacy of immunotherapies to clear existing amyloid plaques from the brains of individuals with AD. Gantenerumab is a humanized anti-Aβ monoclonal antibody that recognizes Aβ fibers [97]. Roche currently has a phase I/II trial ongoing with a redesigned version of gantenerumab called trontinemab. Trontinemab is gantenerumab linked to Roche’s “brain shuttle” technology to enhance brain delivery via the TfR. Clinical trial results show that Trontinemab is safe at high doses [98]. At present, Trontinemab is in a Phase I trial that began evaluating multiple doses in 120 people with prodromal or mild-to-moderate AD and a positive amyloid positron emission tomography (PET) scan. Aducanumab is a high-affinity human monoclonal antibody against Aβ that selectively binds to Aβ aggregates and does not bind to Aβ monomers [84]. After intravenous injection, Aducanumab preferentially binds to Aβ in the peripheral blood and brain parenchyma rather than to Aβ on the vascular walls [92]. In a phase III clinical trial, the AD patients treated with Aducanumab showed a 22% reduction in CDR-SB score decline fom baseline compared with the placebo group in the 78th week [99].

The binding strength of Lecanemab to 75–300 kDa Aβ fibers was 100 times higher than that of Aducanumab, and the binding strength to 300–500 kDa Aβ fibers was 25 times higher than that of Aducanumab [100]. The clinical cognitive impairment scores of patients taking Lecanemab (27%) were 4% higher than those of individuals taking Aducanumab (23%) [100].

### Challenges in clinical trials for passive immunity

#### BBB penetration

**Fig. 5:**
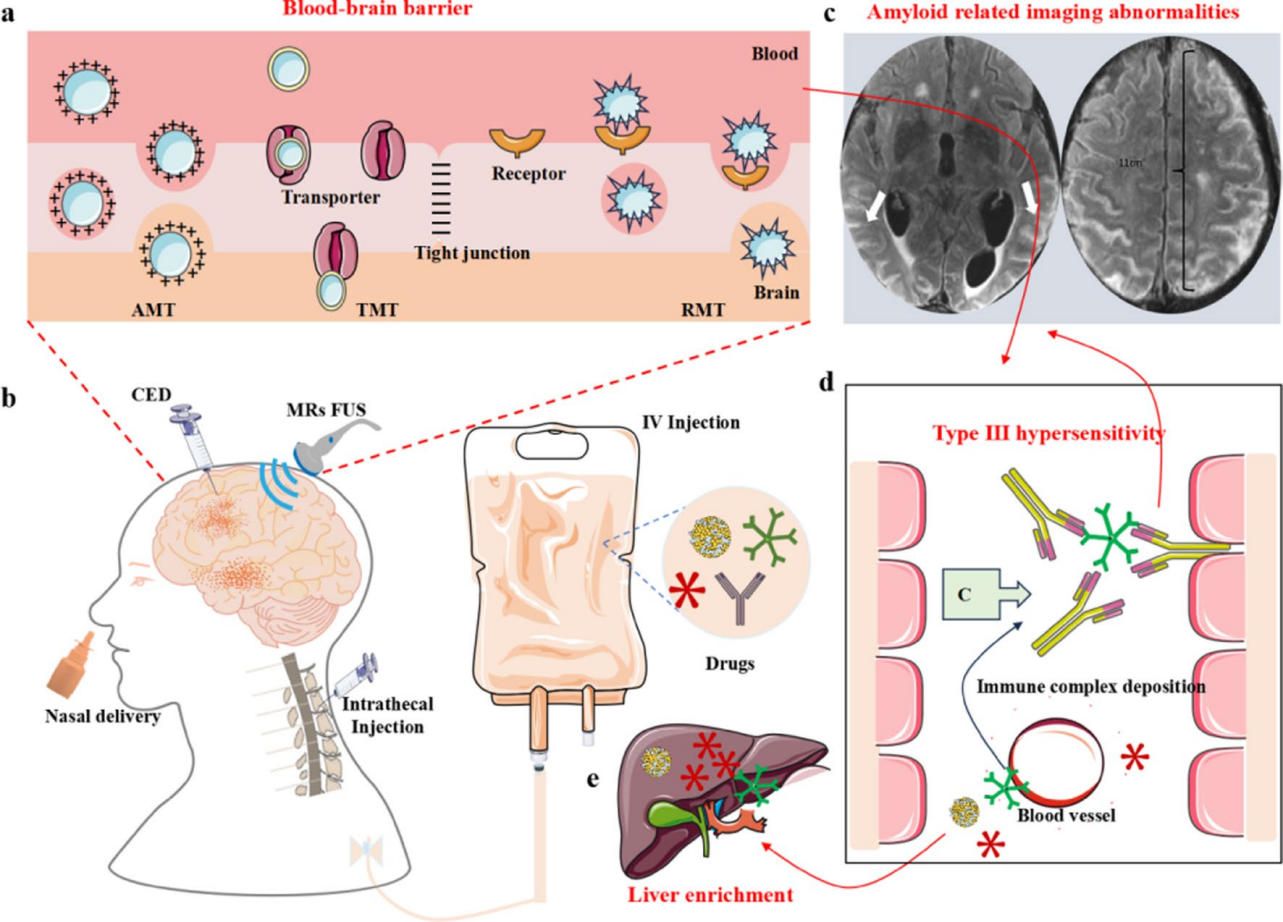
Strategies and shortcomings of passive immunotherapy. **a** The drugs are mainly enriched in the brain by crossing the BBB through adsorptive-mediated transcytosis (AMT), transporter-mediated transcytosis (TMT), and receptor-mediated transcytosis (RMT). **b** Methods of drug administration to improve drug entry in the brain. The methods include nasal delivery, convection-enhanced delivery (CED), magnetic resonance-guided focused ultrasound (MRs FUS), intrathecal injection, and intravenous (IV) injection. **c** Most antibodies induce amyloid-related imaging abnormalities in clinical trials. **d** Antibody-mediated type III hypersensitivity reactions. **e** Intravenous injection of antibodies or other drugs via liver metabolism

For antibodies that are used to specifically clear Aβ, only 0.1% of them can enter the brain after intravenous injection [101–103]. The poor penetration of the BBB by anti-Aβ antibodies results in an antibody level in the brain lower than the concentration needed to continuously inhibit the formation of Aβ aggregates or effectively degrade and clear Aβ. Therefore, improved efficiency of antibody entry in the brain is required to enhance Aβ clearance. Currently, there are three main methods for delivering brain-targeted drugs (Fig. 5a, b). (1) Bypassing the BBB to deliver drugs to the brain, which mainly includes invasive and non-invasive administration. The invasive routes of administration (intraparenchyma administration, convection-enhanced delivery, and intrathecal administration) are performed primarily using specific devices that directly penetrate the skull or lumbar spine [104]. The non-invasive administration is typically performed directly via nasal administration. (2) Enhancing the permeability of the BBB. This method mainly includes high heat [105], high permeability, and focused ultrasound [106]. However, when the BBB is opened, the effect is local, temporary, and non-invasive, and the degree of BBB opening and the amount of medication administered are challenging to evaluate. (3) Crossing the BBB. Receptor-mediated transcytosis is one of the most common strategies for BBB crossing, through TfR, insulin receptors (IR), and LRP-1. The anti-TfR antibody 8D3 is the most commonly used method [107–109].

#### Low complement response

Although antibodies are well tolerated in vivo, they may also be recognized as a foreign component by the recipient, thus causing an adverse immune response. The adverse reactions caused by peripheral injection of antibodies can be classified into hypersensitivity types I, II, and III according to their pathogenesis [110]. Among them, the type I hypersensitivity is mostly mediated by immunoglobulin E (IgE) antibodies, which react quickly and usually cause physiological function disruption [111]. The type II hypersensitivity is mainly caused by IgG or IgM, which causes hemolysis with the participation of macrophages, natural killer cells, and the complement system [111]. The type III hypersensitivity is mainly caused by the deposition of soluble immune complexes formed by antibodies and antigens in blood vessels, which causes local necrosis, tissue hyperemia, and edema by activating the complement system [111, 112] (Fig. 5d).

Amyloid-related imaging abnormalities (ARIA), including ARIA-edema/effusion (ARIA-E) and ARIA-hemosiderosis/microhemorrhages (ARIA-H), may occur, often during early anti-Aβ monoclonal antibody treatment [113]. In the phase III study of Aducanumab in AD patients, ARIA occurred in 425 (41.3%) of 1029 patients in the 10 mg/kg group [114]. Similarly, in the phase III clinical trial of Lecanemab in AD patients, 12.6% of AD patients developed ARIA, and 17.3% of AD patients had intracranial hemorrhage [114] (Fig. 5c). ARIA is caused by a classical complement reaction activated by antibodies. Therefore, the antibody-mediated adverse immune response is a critical factor that limits the efficacy of passive immunotherapy.

#### High liver enrichment

Recent studies have shown that AD is associated with abnormal liver function [115]. Anti-Aβ antibodies and antibody-modified nanoparticles are enriched by approximately 33% in the liver after intravenous injection, which will mediate adverse immune reactions and increase the liver burden [116] (Fig. 5e). Moreover, neurotoxic Aβ in the liver also induces and accelerates the pathogenesis of AD [117]. Therefore, in AD immunotherapy, when clearing brain Aβ, reducing the non-specific enrichment of antibodies in the liver is an urgent issue to be addressed [118].

#### Lower specificity

In AD, Aβ plaques in the brain continuously recruit Aβ monomers and oligomers to form larger plaques [24, 119]. Aβ oligomers are considered to be the main form of neurotoxicity, and Aβ toxicity is negatively correlated with the degree of aggregation [25]. Therefore, an excellent anti-Aβ antibody should have high specificity for soluble Aβ and exhibit strong and continuous inhibition and depolymerization of Aβ aggregates.

#### Systemic diseases

AD may be a heterogeneous disorder with involvement of biological and psychosocial factors, including immune system dysfunction, hepatic dysfunction, renal insufficiency and diabetes mellitus [120]. First, the immune system dysfunction is considered the most crucial pathological factor in AD. Microglia and macrophages express the class A1 scavenger receptors (Scara1) to bind and phagocytose fibrillar Aβ aggregates. Mononuclear phagocytes also express several Aβ-degrading enzymes, such as insulin-degrading enzyme and neprilysin. In AD, the expression of these phagocytic Aβ receptors and Aβ-degrading enzymes decreases significantly in microglia, resulting in reduced Aβ phagocytosis [121, 122]. In addition, chronic systemic inflammation, such as rheumatoid arthritis and periodontitis, can accelerate the development of AD [123]. Second, the liver is the body’s most crucial organ for protein synthesis and metabolism. Peripheral Aβ is degraded directly in the hepatocytes or indirectly via liver-mediated albumin and Aβ-associated lipoproteins [61]. Studies have shown that the elevated aspartate transaminase/alanine transaminase (ALT) ratio and lower ALT levels are associated with AD pathology (Aβ, Tau) in the brain and poor cognitive performance in participants [124]. Therefore, liver dysfunction is closely related to the pathogenesis of AD. In addition, ApoE is synthesized and secreted in the liver, which regulates Aβ clearance by transport across the BBB, enzymatic degradation, and other pathways [125]. Third, the kidney plays a critical role in the peripheral clearance of Aβ, and soluble Aβ is a normal component of human urine [126]. Clinical studies have shown that patients with chronic kidney disease are more susceptible to cognitive impairment and abnormal deposition of Aβ in the brain [66]. Fourth, diabetes affects the development of AD probably through the disruption of Aβ metabolism in the brain and the periphery [127]. In people with diabetes, excess insulin inhibits IDE-mediated Aβ degradation. In addition, Aβ clearance is also impaired by other mechanisms such as oxidative stress, BBB dysfunction, activation of inflammatory pathways, and hypercholesterolemia [128–130].

## Conclusion and perspectives

Insoluble Aβ plaques act as a reservoir for soluble Aβ, and the potential removal of insoluble Aβ from the brain could offer several advantages by eliminating all toxic forms of Aβ (oligomers and fibrils) compared to the removal of soluble Aβ. Currently, passive immunotherapy with intravenous monoclonal anti-Αβ antibodies is a promising strategy for AD treatment to remove neurotoxic insoluble Aβ from the brain via the peripheral system. However, this method faces challenges such as antibody-mediated adverse immune responses, limited efficiency in brain penetration, and significant non-specific accumulation in the liver.

The fundamental goal of AD research is to stop and eventually cure the disease. A better understanding of the pathophysiology of AD is critical for AD treatment. Aβ peptides are metabolized (anabolized and catabolized) in both the brain and peripheral tissues. Significantly, abnormal metabolism of Aβ peptides which could communicate bidirectionally between the two regions, could cause both central and systemic abnormalities, which in turn can generate feedback loops. Indeed, this close interaction between the brain and periphery, particularly about Aβ peptide metabolism, offers new insights into the pathogenesis of AD. Therefore, AD can be considered both a disease of the brain and a systemic disease. Understanding the pathophysiology of AD beyond the CNS is essential for the use of different therapeutic approaches or multi-target therapies, learning from unsuccessful clinical trials with Aβ and non-amyloid-based approaches (metabolic and anti-tau therapies). However, clarifying how peripheral processes influence AD pathogenesis, determining the interactions between the brain and the peripheral systems during AD progression, and investigating plasma Aβ as a blood-based biomarker for AD diagnosis, remain challenging. Future studies should explicitly consider systemic therapeutic strategies for prevention, including (1) improving peripheral Aβ peptide clearance (enhancing phagocytosis, proteolytic degradation, and excretion), (2) identifying and managing systemic abnormalities or developing a comprehensive strategy that targets both brain and peripheral abnormalities, and (3) developing rejuvenation factors/Aβ-peptide sequestrants in the blood for systemic rejuvenation therapies. In summary, this review provides new insights into the pathogenesis of AD and potential diagnostic and therapeutic advances.

**Fig. 6:**
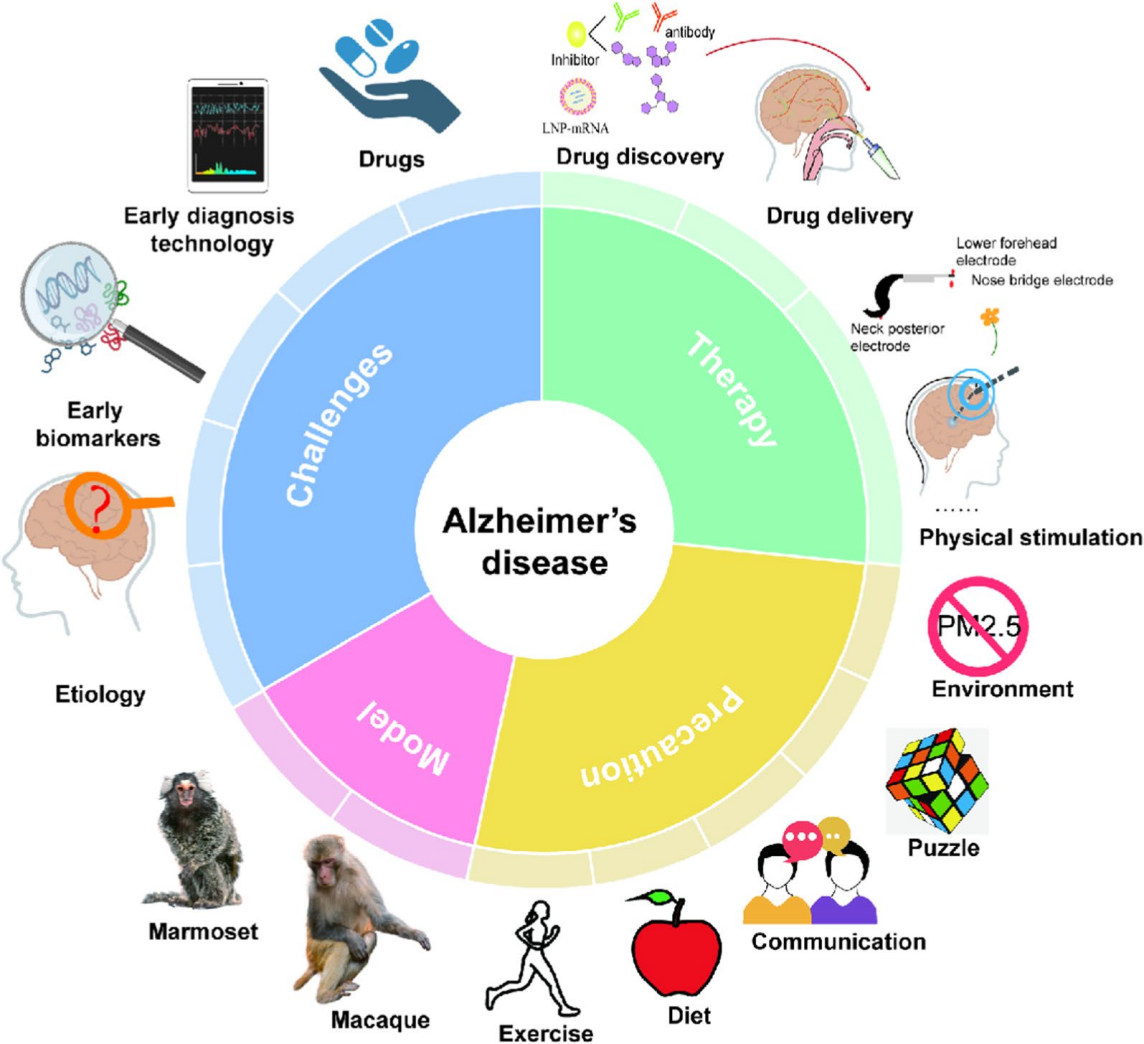
The prospects of AD prevention and therapy. Challenges remain in understanding AD etiology and pathologic mechanisms, finding early biomarkers, and developing early diagnostic techniques and effective drugs. Promising research directions for AD treatment include model establishing, drug discovery and delivery, and physical stimulation. Preventive strategies focus on lifestyle modifications, including improving the living environment, playing puzzle games, promoting communication with others, maintaining a healthy diet, and physical exercise

AD still faces three significant challenges (Fig. 6). First, the etiology of the disease remains unclear and hinders scientific prevention efforts. Second, there is a need for more simple and reliable methods for early diagnosis. Current clinical diagnosis relies heavily on neuropsychological tests and imaging techniques. Due to the low accuracy of cognitive assessments and the high cost of brain imaging, most AD patients receive a diagnosis at late stages. Third, the lack of effective therapeutic drugs complicates treatment and rehabilitation. Early prevention and treatment are crucial for comprehensive improvement of AD.

In terms of prevention, research has shown that specific strategies can reduce the risk or delay the onset of the disease (Fig. 6). (1) Physical activity can improve blood flow to the brain and promote overall well-being [131]. (2) A healthy diet rich in fruits, vegetables, whole grains, lean proteins, and healthy fats is recommended. Some studies suggest that following the Mediterranean diet can positively impact brain health [111]. (3) Mentally stimulating activities such as playing games, reading, learning new skills, or practicing hobbies can keep the brain active and improve cognitive health. A large-scale clinical trial, the Advanced Cognitive Training for Independent and Vital Elderly (ACTIVE) study, has shown that cognitive training improves the cognitive performance of healthy older adults aged 65 and over [132]. (4) Managing chronic conditions like high blood pressure, diabetes, obesity, and high cholesterol can help prevent or delay AD [133]. (5) Exposure to polluted air, especially PM_2.5_, can lead to inflammation, oxidative stress, and damage to blood vessels, which can affect brain health and potentially contribute to neurodegenerative diseases [134, 135]. Therefore, limiting exposure to polluted air can reduce the risk of AD.

Although currently there is no cure for AD, there are several therapeutic strategies and interventions aimed at alleviating symptoms and improving the general well-being of patients (Fig. 6). Among them, FDA-approved cholinesterase inhibitors and anti-Aβ antibodies are effective in treating emotional and cognitive symptoms in patients, although they may not halt or reverse the progression of AD. There is ongoing research on the use of mRNAs to stimulate specific proteins or molecules in the brain, using lipid nanoparticles to encapsulate mRNA molecules and deliver them into cells to produce target proteins [136]. Drug delivery methods are critical for treatment efficacy. Researchers are investigating nanotechnology to develop nanoparticles that can cross the BBB and deliver drugs directly to the brain. These nanoparticles can improve drug solubility and stability and increase brain uptake [137]. In addition, light [138], sound [139], and gamma wave [140] are increasingly used as non-invasive therapies to improve cognitive function. The efficacy and safety of these therapies for AD need to be thoroughly investigated in well-designed clinical trials. In addition, research is underway to develop various models to understand, diagnose, and treat AD, using biological, animal, cellular, and computational approaches. Each model has its strengths and weaknesses [141] (Fig. 6). Much work is being done to characterize animal models of AD, from rodents to primates, to improve our understanding of the pathophysiology of the disease and to find models suitable for studying potential treatments.

## Data Availability

Not applicable.

## Abbreviations

AD: Alzheimer’s disease
Aβ: Amyloid-beta
APP: Amyloid precursor protein
CNS: Central nervous system
BBB: Blood–brain barrier
CSF: Cerebrospinal fluid
LRP-1: Low density lipoprotein receptor related protein-1
ApoE: Apolipoprotein E
IL-1β: Interleukin-1β
IL-6: Interleukin-6
iNOS: Inducible nitric oxide synthase
TREM2: Triggering receptor expressed on myeloid cells-2 receptor2
Tyrob: Tyrosine protein–protein binding
NF-κB: Nuclear factor κB
NLRP3: Nucleotide binding oligomerization domain-like receptor protein 3
ROS: Reactive oxygen species
TLR4: Toll-like receptor 4
TfR: Transferrin receptors
ARIA: Amyloid related imaging abnormalities
